# Immunological evidence for in vivo production of novel advanced glycation end-products from 1,5-anhydro-D-fructose, a glycogen metabolite

**DOI:** 10.1038/s41598-019-46333-2

**Published:** 2019-07-15

**Authors:** Akiko Sakasai-Sakai, Takanobu Takata, Hirokazu Suzuki, Ikuro Maruyama, Yoshihiro Motomiya, Masayoshi Takeuchi

**Affiliations:** 1 Department of Advanced Medicine, Medical Research Institute, Kanazawa Medical University, Uchinada-machi, Ishikawa, 920-0293 Japan; 2 Department of Organic and Medicinal Chemistry, Faculty of Pharmaceutical Sciences, Hokuriku University, Kanazawa, Ishikawa 920-1181 Japan; 3 Systems Biology in Thromboregulation, Kagoshima University Graduate School of Medical and Dental Sciences, Kagoshima, 890-8520 Japan; 4 Suiyukai Clinic, Kashihara Nara, 634-0007 Japan

**Keywords:** Biochemistry, Immunology

## Abstract

The anhydrofructose pathway is an alternate pathway for glycogen degradation by α-1,4-glucan lyase. The sugar 1,5-anhydro-D-fructose (1,5-AF) acts as the central intermediate of this pathway, but its physiological role of in mammals is unclear. Glycation reactions forming advanced glycation end-products (AGEs) are important in the development of complications of diabetes mellitus. We hypothesized that 1,5-AF may contribute to cellular damage by forming 1,5-AF-derived AGEs (AF-AGEs) with intracellular proteins. To clarify the role of 1,5-AF in protein modification, we created a novel antibody targeting AF-AGEs. Serum albumin modified by AF-AGEs was prepared by incubating rabbit serum albumin (RSA) or bovine serum albumin (BSA) with 1,5-AF. After immunizing rabbits with AF-AGEs-RSA, affinity chromatography of anti-AF-AGE antiserum was performed on a Sepharose 4B column coupled with AF-AGEs-BSA or N-(carboxymethyl)/N-(carboxyethyl)lysine-BSA. A novel immunopurified anti-AF-AGE antibody was obtained and was characterized using a competitive enzyme-linked immunosorbent assay. Then an AF-AGEs assay was established using this immunopurified antibody. This assay was able to detect AF-AGEs in human and animal serum samples. Finally, intracellular accumulation of AF-AGEs was shown to be associated with damage to cultured hepatocytes (HepG2 cells). This is the first report about *in vivo* detection of AF-AGEs with a novel structural epitope.

## Introduction

**Figure 1:**
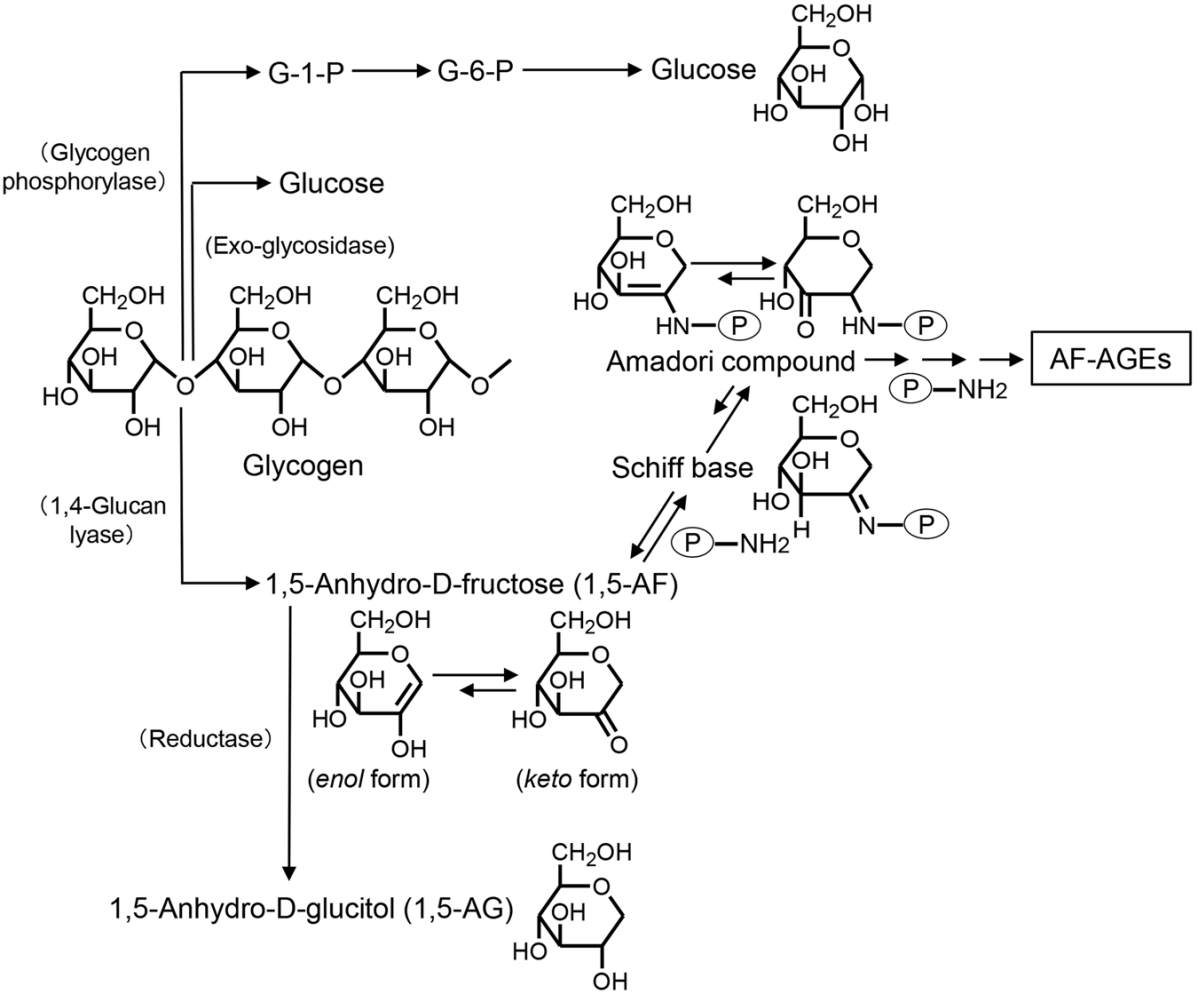
Pathway for formation of 1,5-anhydro-D-fructose (1,5-AF), a metabolite of glycogen, and 1,5-AF-derived AGEs (AF-AGEs). Glycogen is degraded to glucose-1-phosphate (G-1-P) by glycogen phosphorylase, and to glucose by exo-glycosidase. α-1,4-Glucan lyase produces 1,5-AF by the elimination-mediated degradation of glycogen. 1,5-AF is metabolized to 1,5-anhydro-D-glucitol (1,5-AG) by reductase. Non-enzymatic glycation reactions between 1,5-AF and the ε-amino groups of lysine residues of proteins (or the guanidino groups of arginine residues or the N-terminal α-amino groups of proteins) initially form reversible Schiff base adducts. These Schiff base adducts then slowly undergo Amadori rearrangement to produce more stable, but still slowly reversible, adducts. Subsequently, the early glycation products undergo further complex reactions, such as rearrangement, dehydration, and condensation, to become irreversibly cross-linked, heterogeneous AGEs. This end-stage glycation process is still only partially characterized. G-1-P, glucose-1-phosphate; G-6-P, glucose-6-phosphate; AF-AGEs, 1,5-anhydro-D-fructose-derived AGEs; P-NH_2_, free amino residues of proteins.

In mammals, including humans, glucose is stored as glycogen to provide energy. When energy is needed, glycogen is degraded to glucose-1-phosphate by glycogen phosphorylase, and to glucose by exo-glycosidase (Fig. 1). Yu *et al*. identified an alternate pathway for glycogen catabolism^1^, the anhydrofructose (AF) pathway^2^, which forms secondary metabolites from glycogen via 1,5-anhydro-D-fructose (1,5-AF). 1,5-AF is metabolized to 1,5-anhydro-D-glucitol (1,5-AG) by reductase^2^ (Fig. 1). Although information about the physiological role of 1,5-AF and 1,5-AG in glucose homeostasis in humans is limited, the serum concentration of 1,5-AG differs between healthy individuals and patients with diabetes, leading to use of 1,5-AG a marker of diabetes control^3,4^. However, the physiological role of 1,5-AF in mammals remains unclear. 1,5-AF is a unique keto-monosaccharide, in which the tautomers of its ketone, enol, enediol, and hydrated forms exist in equilibrium^1,5,6^. Hemiacetal bonding of the carbonyl group does not occur during its formation and it is fully hydrated in aqueous solution, suggesting an active role in metabolism^1,5,6^.

Advanced glycation end-products (AGEs) are produced as a result of non-enzymatic glycation reactions between ketone or aldehyde groups of reducing sugars, including glucose or fructose, and the ε-amino group of lysine residues, the guanidino group of arginine residues, or the N-terminal α-amino groups of proteins^7–10^. AGEs formation is suggested to be accelerated *in vivo* by conditions such as hyperglycemia and aging^7–10^. Although elevation of the glucose level was previously considered to play a primary role in the glycation reaction, glucose is one of the least reactive sugars in biological systems^11^. In fact, AGEs formation actually depends on various non-glucose metabolites, including trioses and dicarbonyl compounds, which are mainly intracellular and participate in glycation at a much faster rate than glucose^10,12–15^. 1,5-AF is a novel metabolic intermediate of glycogen, and 1,5-AF-derived AGEs (AF-AGEs) are expected to largely accumulate in hepatocytes because the liver is the chief site of glycogen metabolism. The initial phase of the glycation reaction involving 1,5-AF is condensation of its carbonyl group with amino groups of proteins (Fig. 1), and is similar to the reaction for glucose/fructose^15,16^. 1,5-AF is thought to be more important for AGEs formation than glucose and fructose because their anomerization equilibrium is shifted toward the reactive open chain forms of sugars. Although *in vivo* formation of AF-AGEs has been postulated, confirmatory evidence has not been obtained.

In the present study, we created a novel antibody targeting AF-AGEs from rabbit serum albumin (RSA) and investigated its features. We also obtained the first evidence that a particular AF-AGE epitope causes specific cell damage in the HepG2 human hepatocellular carcinoma (HCC) cell line, and detected this AF-AGEs in human and animal serum specimens.

## Results

### Characterization of anti-AF-AGE antiserum and isolation of an anti-AF-AGE antibody

**Figure 2:**
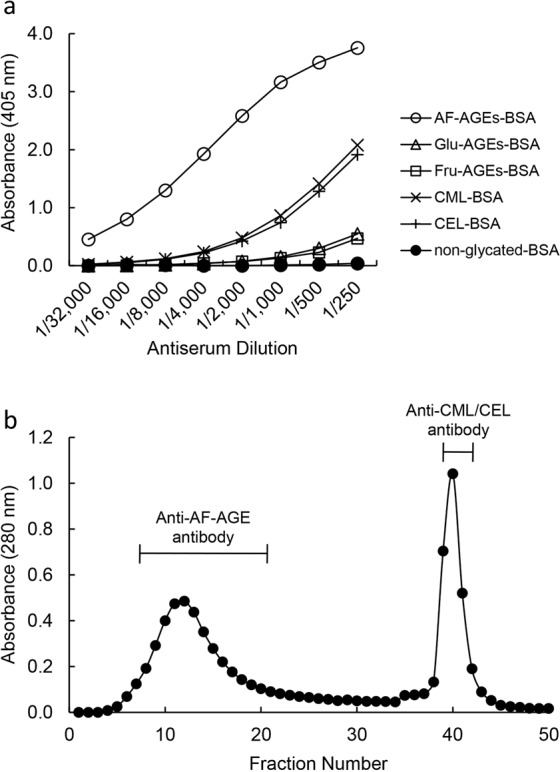
Immunoreactivity of anti-AF-AGE antiserum and separation of the anti-AF-AGE antibody by CML-/CEL-BSA affinity chromatography. **(a)** The immunoreactivity of anti-AF-AGE antiserum with AF-AGEs-BSA, glucose-derived AGEs (Glu-AGEs-BSA), fructose-derived AGEs (Fru-AGEs-BSA), N-(carboxymethyl)lysine-BSA (CML-BSA), N-(carboxyethyl)lysine-BSA (CEL-BSA), and non-glycated BSA was assessed by non-competitive ELISA using various concentrations of anti-AF-AGE antiserum. **(b)** Separation of the anti-AF-AGE antibody from anti-AF-AGE antiserum by CML-/CEL-BSA affinity chromatography. Affinity chromatography was performed as described in Materials and Methods.

We obtained anti-AF-AGE antiserum from rabbits immunized with AF-AGEs-RSA. Figure 2 shows the reactivity of this anti-AF-AGEs-RSA antiserum with AF-AGEs-bovine serum albumin (AF-AGEs-BSA), glucose-derived AGEs (Glu-AGEs-BSA), fructose-derived AGEs (Fru-AGEs-BSA), N-(carboxymethyl)lysine-BSA (CML-BSA), N-(carboxyethyl)lysine-BSA (CEL-BSA), and non-glycated BSA in a non-competitive enzyme-linked immunosorbent assay (ELISA). The antiserum reacted with AF-AGEs-BSA, but not with Glu-AGEs-BSA, Fru-AGEs-BSA or non-glycated BSA incubated without 1,5-AF (Fig. 2a). Cross-reactivity studies showed that this antiserum reacted weakly with CML-BSA or CEL-BSA. Therefore, the antiserum appeared to contain a specific antibody targeting AF-AGEs and also an antibody for CML/CEL (Fig. 2a). Degradation of Amadori products leads to creation of CML^17^ and CEL is a homologue of CML. The antiserum was passed through an affinity column coupled with AF-AGEs-BSA in order to obtain a purified anti-AF-AGE antibody, and then was subjected to further separation by CML-/CEL-BSA affinity chromatography (Fig. 2b). The amount of antibody binding to the CML-/CEL-BSA affinity gel (eluted as the second peak) was calculated as a percentage of the unbound antibody (eluted as the first peak), revealing that bound anti-CML/CEL antibody accounted for approximately 35% of total antibodies in the antiserum.

### Specificity of the immunopurified anti-AF-AGE antibody

**Figure 3:**
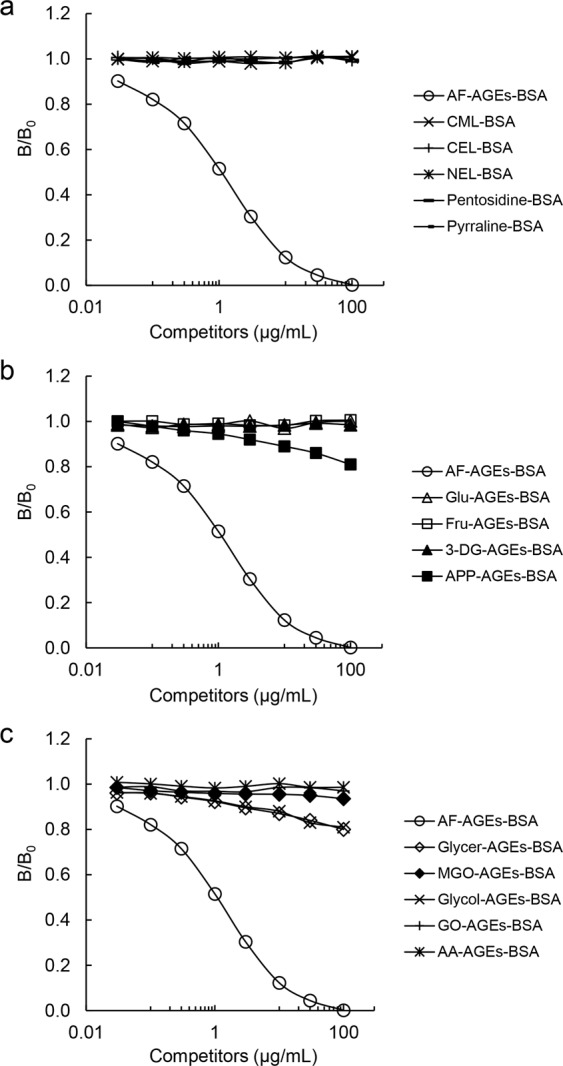
Immunoreactivity of the immunopurified anti-AF-AGE antibody with various AGE proteins. (**a**–**c**) The anti-AF-AGE antibody obtained by CML-/CEL-BSA affinity chromatography was characterized by performing competitive ELISAs with several AGE-modified proteins. CML-BSA, N-(carboxymethyl)lysine-BSA: CEL-BSA, N-(carboxyethyl)lysine-BSA; NEL-BSA, N-(ethyl)lysine-BSA. Glu-AGEs-BSA, glucose-derived AGEs; Fru-AGEs-BSA, fructose-derived AGEs; 3-DG-AGEs-BSA, 3-deoxyglucosone-derived AGEs; APP-AGEs-BSA, ascopyrone P-derived AGEs; Glycer-AGEs-BSA, glyceraldehyde-derived AGEs; MGO-AGEs-BSA, methylglyoxal-derived AGEs; Glycol-AGEs-BSA, glycolaldehyde-derived AGEs; GO-AGEs-BSA, glyoxal-derived AGEs; AA-AGEs-BSA, acetaldehyde-derived AGEs.

The immunopurified anti-AF-AGE antibody was used to perform competitive ELISAs with various AGE proteins. To clarify whether this antibody recognized previously characterized AGEs, testing was done with CML-BSA, CEL-BSA, N-(ethyl)lysine-BSA (NEL-BSA), pentosidine-BSA, and pyrraline-BSA. None of these AGEs inhibited binding of the immunopurified anti-AF-AGE antibody to AF-AGEs-BSA (Fig. 3a). We then investigated whether C-6 compound-derived AGEs, such as Glu-AGEs-BSA, Fru-AGEs-BSA, 3-deoxyglucosone-derived AGEs (3-DG-AGEs-BSA), and AGEs derived from ascopyrone P (a secondary metabolite of 1,5-AF in fungi^18^/heating product of 1,5-AF^19^: APP-AGEs-BSA) bound to the anti-AF-AGE antibody. As a result, none of these compounds inhibited binding of the immunopurified anti-AF-AGE antibody to AF-AGEs-BSA (Fig. 3b). We also investigated whether the immunopurified anti-AF-AGE antibody reacted with C-3/C-2 compounds, such as AGEs derived from glyceraldehyde (Glycer-AGEs-BSA), methylglyoxal (MGO-AGEs-BSA), glycolaldehyde (Glycol-AGEs-BSA), glyoxal (GO-AGEs-BSA), and acetaldehyde (AA-AGEs-BSA). Once again, none of these compounds inhibited binding of the immunopurified anti-AF-AGE antibody to AF-AGEs-BSA (Fig. 3c). Collectively, these results indicate that AF-AGEs-BSA exhibited its strongest reactivity with the immunopurified anti-AF-AGE antibody, and the antibody also specifically recognized unknown AF-AGE structures.

### Detection of AF-AGEs in human/animal sera

The presence of AF-AGEs in commercially available human/rat/mouse serum was investigated by performing competitive ELISA with the immunopurified anti-AF-AGE antibody and an AF-AGEs-BSA standard. The amount of AF-AGEs in two lots of sera was as follows: 5.88 ± 0.38 and 7.66 ± 0.15 U/mL (human), 22.11 ± 0.84 and 17.05 ± 1.33 U/mL (rat), and 18.73 ± 0.95 and 14.23 ± 0.79 U/mL (mouse). Thus, the immunopurified anti-AF-AGE antibody could detect AF-AGEs in human or animal serum.

### Induction of HepG2 cell injury by intracellular AF-AGEs

**Figure 4:**
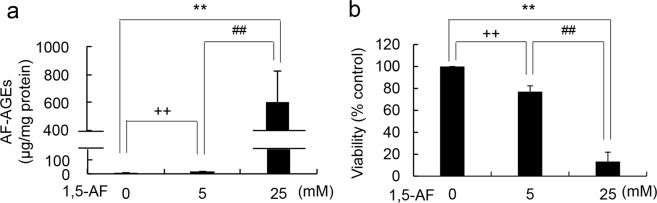
Incubation of hepatocytes with 1,5-AF caused accumulation of intracellular AF-AGEs and cell damage. **(a)** Slot blot (SB) analysis of intracellular AF-AGEs. Cell extracts were prepared from HepG2 cells treated with 0, 5, or 25 mM 1,5-AF for 72 h. The amount of AF-AGEs was calculated from a standard curve prepared with AF-AGEs-BSA. SB analysis was performed three times independently and data are shown as the mean ± S.D. (N = 3). P values were calculated by Tukey’s test or Student’s t-test. **p < 0.01 vs. 0 mM 1,5-AF by Tukey’s test. ^++^p < 0.01 vs. 0 mM 1,5-AF by Student’s t-test. ^##^p < 0.01 vs. 5 mM 1,5-AF by Student’s t-test. (**b**) Cell viability was assessed by the CellTiter-Glo assay. HepG2 cells were incubated for 72 h with 0, 5, or 25 mM 1,5-AF in triplicate. Three independent experiments were performed and data are shown as the mean ± S.D. (N = 3). P values were calculated by Tukey’s test. **p < 0.01 vs. 0 mM 1,5-AF, ^++^p < 0.01 vs. 0 mM 1,5-AF, ^##^p < 0.01 vs. 5 mM 1,5-AF.

AF-AGEs should accumulate in hepatocytes because the liver is the main site of glycogen metabolism. We used the HepG2 human HCC cell line to assess the effects of intracellular AF-AGEs. In humans, the serum 1,5-AG concentration was reported to be 0.032–0.367 mM^20^, and the rate of 1,5-AF incorporation is approximately two orders of magnitude slower than that of 1,5-AG incorporation^21,22^. Accordingly, exposure to 1,5-AF at the millimolar level (5–25 mM) is considered to be an appropriate concentration for cultured cells. To clarify whether accumulation of AF-AGEs occurs in hepatocytes treated with 1,5-AF, HepG2 cells were incubated with 0–25 mM 1,5-AF and cell extracts were subjected to slot blotting (SB) with the immunopurified anti-AF-AGE antibody to detect intracellular AF-AGEs. Accumulation of intracellular AF-AGEs showed a dose-dependent increase with 1,5-AF treatment (Fig. 4a & Supplementary Figs S1a, S2). Next, we investigated the cytotoxicity of intracellular AF-AGEs, revealing that 1,5-AF caused dose-dependent damage to HepG2 cells (Fig. 4b & Supplementary Fig. S1b). Cytotoxicity of 1,5-AF for HepG2 cells was blocked by preincubation with 4 mM aminoguanidine, an inhibitor of AGE formation (cell viability: 106 ± 5.5% with 5 mM 1,5-AF, 87.0 ± 15.0% with 10 mM 1,5-AF, n = 6). It is thought that 1,5-AF undergoes reduction to 1,5-AG by reductase in hepatocytes. No decrease of cell viability (103 ± 6.7%, n = 9) was observed after adding 5 mM 1,5-AG to HepG2 cells, and there was no significant decrease of cell viability (85.0 ± 10.1%, n = 9) even when 25 mM 1,5-AG was added. These findings suggested that intracellular accumulation of AF-AGEs causes injury to human hepatocytes.

## Discussion

The AF pathway is an alternate glycogen-degrading pathway that was identified by Yu *et al*. and established by the International Union of Biochemistry and Molecular Biology (IUBMB) in 2006^2^. α-1,4-Glucan lyase (EC 4.2.2.13) produces 1,5-AF via degradation of glycogen^2^ (Fig. 1). This pathway operates under conditions of biotic and abiotic stress in fungi as well as in red algae, and it may be part of the response to starvation and have a role in signal transduction in other microorganisms^23^. In humans and other mammals, the functions of metabolites produced by this pathway are still unclear, although 1,5-AF and its metabolite 1,5-AG may be involved in regulating glycogen metabolism^24^. 1,5-AG is a monosaccharide with a similar structure to glucose (Fig. 1), and it can be used as an indicator of short-term glycemic excursions, particularly for monitoring postprandial glycemic control^25,26^. 1,5-AG reflects changes in glycemic control over a period of 1 to 2 weeks^27^. The normal plasma level of 1,5-AG is approximately 14 μg/mL, whereas it falls to approximately 2 μg/mL in type 2 diabetes mellitus^26^. Accordingly, 1,5-AG can be employed to monitor glycemic control in patients with diabetes^3,4,26,28^. On the other hand, the concentration of 1,5-AF, the precursor of 1,5-AG, in human body fluids and inside cells is unclear.

1,5-AF is a keto-monosaccharide that also shares structural similarity with glucose (Fig. 1). It is possible that 1,5-AF may have an important role in intracellular glycation because it is produced by hepatocytes along with 1,5-AG. While 1,5-AG is not involved in glycation reactions because it does not have a ketone/aldehyde (Fig. 1) and it does not cause ring opening by anomerization, similar to glucose and fructose, we suggest that 1,5-AF could contribute to AGE formation due to its higher reactivity compared with glucose or fructose. By contributing to AF-AGEs production, 1,5-AF may cause alterations of various proteins, leading to hepatocyte and dysfunction damage.

**Figure 5:**
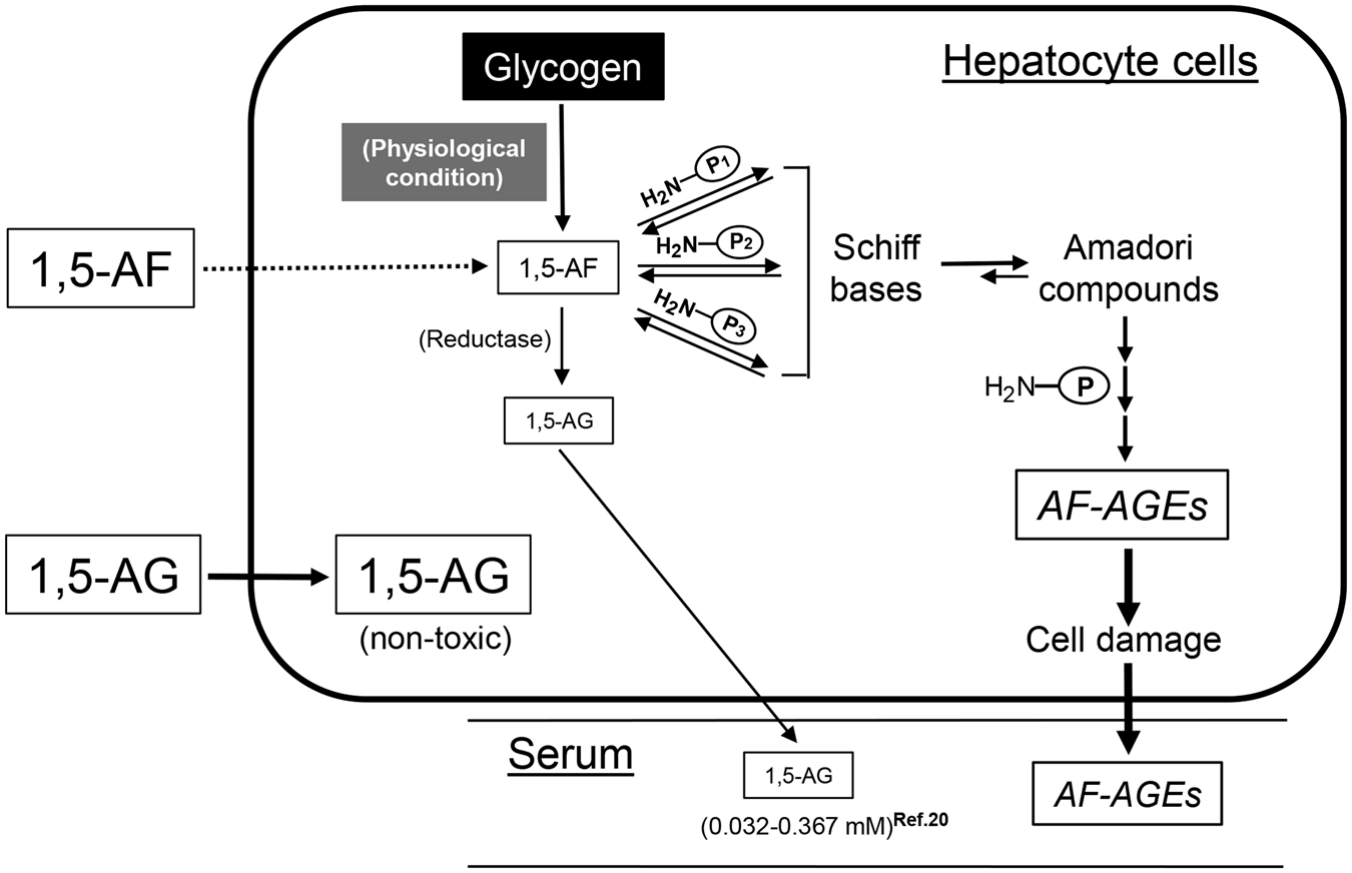
Proposed mechanism for the effects of AF-AGEs on hepatocytes. 1,5-AF produced by hepatocytes reacts non-enzymatically with the ε- or α-amino groups of intracellular proteins to form Schiff bases and then Amadori products. Early glycation products undergo further complex reactions, such as rearrangement, dehydration, and condensation, to become irreversibly cross-linked, heterogeneous AF-AGEs. Accumulation of AF-AGEs damages hepatocytes and these AGEs then leak into the blood, increasing the circulating AF-AGE level. 1,5-AF, 1,5-anhydro-D-fructose; 1,5-AG, 1,5-anhydro-D-glucitol; AF-AGEs, 1,5-AF-derived advanced glycation end-products; P-NH_2_, free amino residues of proteins; P_1_-/P_2_-/P_3_-NH_2_, free amino residues of various proteins.

Accordingly, we investigated glycation reactions with a focus on 1,5-AF in the present study (Fig. 1). An immunopurified anti-AF-AGE antibody was produced to detect AF-AGEs (Fig. 2) and an AF-AGEs assay was established by using a competitive ELISA and SB. The immunopurified anti-AF-AGE antibody only recognized AF-AGEs, suggesting that specific AGE structures were formed by incubating the protein with 1,5-AF. The AF-AGEs epitope appears to differ from previously characterized AGE structures, including CML, CEL, NEL, pentosidine, and pyrraline^10,29^ (Fig. 3a), as well as other reducing sugar-/carbonyl compounds-derived AGEs, because the immunopurified anti-AF-AGE antibody did not bind to BSA conjugates of these compounds (Fig. 3b,c). To clarify the role of 1,5-AF in protein modification by glycogen metabolism, we developed a novel antibody targeting AF-AGEs that recognized serum and cellular proteins modified by 1,5-AF and used it to successfully detected AF-AGEs in human/rat/mouse sera. These findings suggest that intracellular accumulation of AF-AGEs may cause liver damage, resulting in leakage of AF-AGEs into the blood from injured cells (Fig. 5).

We also investigated cell damage induced by intracellular AF-AGEs in the HepG2 human HCC cell line by adding 1,5-AF to cultures at millimolar concentrations. K-562 human erythroleukemia cells were reported to take up 1,5-AF, even though 1,5-AF incorporation was very slow, being approximately two orders of magnitude slower than 1,5-AG incorporation^21,22^. The serum 1,5-AG concentrations of healthy subjects from different populations was reported to be 0.032–0.367 mM^20^. Therefore, treatment with 1,5-AF at millimolar concentrations (5–25 mM; predicted intracellular concentration of about 0.05–0.25 mM) was considered to be reasonable for experiments with cultured cells. We demonstrated that 1,5-AF, a precursor of AF-AGEs, induced concentration-dependent damage to cultured HepG2 cells and increased the intracellular AF-AGEs concentration (Fig. 4 & Supplementary Fig. S1). The intracellular level of AF-AGEs, particularly 1,5-AF, was strongly influenced by the activation of secondary glycogen metabolism. AGEs are produced *in vivo* by non-enzymatic glycation, so AF-AGEs formation depends on the turnover of the targets for chemical modification, the time available for the reaction, and the 1,5-AF concentration (Fig. 4a, Supplementary Figs S1a & S2). Even at high concentrations of 1,5-AG (25 mM) approximately two orders of magnitude above its serum concentration, no significant reduction of cell viability was observed. Recently, Ying *et al*. reported that after incubation of HepG2 cells for 2 h with 1,5-AG the intracellular 1,5-AG level was 50–80% of the extracellular level^30^. Furthermore, 1,5-AF-induced cytotoxicity for HepG2 cells was rescued by preincubation with aminoguanidine, an inhibitor of AGE formation. These results suggest that accumulation of AF-AGEs induces human hepatocyte injury, while 1,5-AG and 1,5-AF are not cytotoxic. While these results provide some insight into hepatocyte injury due to AGEs, the mode of cell death and mechanisms induced by AF-AGEs accumulation are still unclear.

In conclusion, the present study is the first to provide information about AF-AGEs in human and animal serum, as well as intracellular AF-AGEs in hepatocytes. Our results suggest that AF-AGEs are generated by hepatocytes via a novel glycogen metabolism pathway and then induce cellular damage (Fig. 5). Further studies are needed to identify AF-AGE-modified proteins and elucidate the mechanisms underlying hepatocyte injury due to AF-AGEs. Although the epitope recognized by the immunopurified anti-AF-AGE antibody was not elucidated in this study, we showed that the antibody target was different from previously well-defined AGEs and other AGEs with unknown structures derived from sugar or carbonyl compounds. Therefore, AF-AGEs seem to be structurally unique, but further spectroscopic and biochemical investigations are needed for confirmation.

## Materials and Methods

### Reagents

1,5-Anhydro-D-fructose (1,5-AF), 1,5-anhydro-D-glucitol (1,5-AG), and ascopyrone P (APP) were kindly provided by Dr. Maruyama. Rabbit serum albumin (RSA), bovine serum albumin (BSA), Dulbecco’s modified Eagle’s medium (D-MEM), fetal bovine serum (FBS), and human/rat/mouse serum samples were obtained from Sigma-Aldrich (MO, USA). A PD-10 column and CNBr-activated Sepharose 4B were purchased from GE Healthcare (Buckinghamshire, England). Centriprep-10 and alkaline phosphatase-linked anti-rabbit IgG were obtained from Millipore Corporation (MA, USA). Diethylenetriamine-pentaacetic acid (DTPA) and 3-[(3-cholamidopropyl)-dimethylammonio]-1-propanesulfonate (CHAPS) were obtained from Dojindo Laboratories (Kumamoto, Japan). Ethylenediamine-N,N,N′,N′-tetraacetic acid (EDTA)-free protease inhibitor cocktail was sourced from Roche Applied Science (Penzberg, Germany). A Bradford method protein assay kit was purchased from Takara Bio, Inc. (Otsu, Japan), while Dc protein assay reagent was from Bio-Rad Laboratories (CA, USA). A CellTiter-Glo assay kit was sourced from Promega (WI, USA), and the horseradish peroxidase (HRP)-linked molecular marker was from Bionexus (CA, USA). HRP-linked goat anti-rabbit IgG antibody was obtained from DAKO (Glostrup, Denmark), and the HepG2human HCC cell line was from ECACC (Salisbury, UK). All other chemicals used were of the highest grade available from commercial sources.

### Preparation of glycated proteins

1,5-AF-derived-AGEs (AF-AGEs-RSA and AF-AGEs-BSA) were prepared as follows. In brief, RSA or BSA (25 mg/mL) was incubated with 0.2 M 1,5-AF and 5 mM DTPA in 0.2 M phosphate buffer (pH 7.4) at 37 °C for 2 weeks under sterile conditions. Then low-molecular-weight reaction products and 1,5-AF were removed by PD-10 column chromatography and dialysis against phosphate-buffered saline (PBS). In addition, AGE-BSAs were prepared as described previously^14,15,29,31^. Briefly, BSA was incubated at 37 °C for 7 days under sterile conditions with 5 mM DTPA in 0.2 M phosphate buffer (pH 7.4) and 0.5 M D-glucose (Glu-AGEs), 0.5 M D-fructose (Fru-AGEs), 0.2 M 3-deoxyglucosone (3-DG-AGEs), 0.2 M ascopyrone P (APP-AGEs), 0.1 M glyceraldehyde (Glycer-AGEs), 0.1 M methylglyoxal (MGO-AGEs), 0.1 M glycolaldehyde (Glycol-AGEs), 0.1 M glyoxal (GO-AGEs), or 0.1 M acetaldehyde (AA-AGEs). In addition, incubation was done under the same conditions for 2 weeks with 3-deoxyglucosone/ascopyrone P and 8 weeks with D-glucose or D-fructose. Then low-molecular-weight reactants and sugars or carbonyl compounds were removed by PD-10 column chromatography and dialysis against PBS. N-(Carboxymethyl)lysine-BSA (CML-BSA), N-(carboxyethyl)lysine-BSA (CEL-BSA), and N-(ethyl)lysine-BSA (NEL-BSA) were prepared by previously described methods^15,29^. Briefly, BSA (50 mg/mL) was incubated for 24 h at 37 °C with glyoxylic acid, pyruvic acid, or acetaldehyde (50 mM each) and sodium cyanoborohydride (150 mM) in 0.2 M phosphate buffer (pH 7.4), after which PD-10 column chromatography was performed with subsequent dialysis against PBS. Protein concentrations were measured with the Dc protein assay reagent using BSA as the standard. Pentosidine-BSA and pyrraline-BSA were provided by MBC (Tokyo, Japan).

### Preparation of anti-AF-AGE antiserum

After AF-AGEs-RSA (4 mg) was emulsified in 50% Freund’s complete adjuvant (Wako Pure Chemical Industries, Ltd., Osaka, Japan), it was injected intradermally in Japanese white rabbits (Sankyo Labo-service Ltd., Tokyo, Japan) once a week intervals for 6 weeks, followed by a 4-mg booster injection after 2 weeks off treatment. Blood samples were collected on the 10^th^ day after the last injection and serum was obtained for purification by affinity chromatography. Immunization of the rabbits and blood collection were performed at Trans Genic Inc., Ltd. (Kobe, Japan).

### Purification of the anti-AF-AGE antibody by affinity chromatography

A specific antibody targeting AF-AGEs was isolated from rabbit antiserum by affinity chromatography. AF-AGEs-BSA or CML-/CEL-BSA (125 mg each) was coupled with CNBr-activated Sepharose 4B (25 mL) according to the manufacturer’s directions and packed into a column (2.5 × 5.5 cm, bed volume: 25 mL). Then 25 mL of anti-AF-AGE antiserum, containing both anti-AF-AGE and anti-CML/-CEL antibodies, was applied to this column. Extensive washing was performed with PBS, after which adsorbed fractions were eluted with 20 mM sodium phosphate buffer that contained 1 M potassium thiocyanate (pH 7.4). Then the eluted fractions were pooled and concentrated using Centriprep-10, after which the concentrated fractions were passed through a PD-10 column equilibrated with PBS. Next, the eluted fraction was applied to a column (1.5 × 6 cm, bed volume: 10 mL) packed with Sepharose 4B coupled to CML-/CEL-BSA, and the column was washed with PBS to obtain the eluted fraction (anti-AF-AGE antibody). Finally, the anti-AF-AGE antibody fractions were pooled and concentrated with Centriprep-10, followed by application to a PD-10 column equilibrated with PBS, before use for ELISA and SB. In contrast, the adsorbed fraction (anti-CML/-CEL antibodies) was eluted with 20 mL of 20 mM sodium phosphate buffer containing 1 M potassium thiocyanate (pH 7.4). Absorbance of the fractions (1.0 mL) was monitored at 280 nm.

### Competitive ELISA

Assessment of ligand inhibition and measurement of AF-AGEs were performed with competitive ELISAs using the immunopurified anti-AF-AGE antibody. Each well of a 96-well enzyme immunoassay/radioimmunoassay plate (flat bottom without a lid, high binding; Corning Incorporated, NY, USA) was coated with 0.75 µg/mL AF-AGEs-BSA standard solution and incubated overnight at 4 °C. Then the wells were washed three times with washing solution (0.3 mL of PBS containing 0.05% Tween-20) and blocking was done by incubation of the plate for 1 h with 0.2 mL of PBS containing 1% BSA. After washing with the washing solution, a test sample (50 µL) was added to each well to compete with the immunopurified anti-AF-AGE antibody (50 µL; 1:3,000). Subsequently, incubation was done for 2 h at 30 °C with gentle shaking on a horizontal rotary shaker, followed by washing with the washing solution and development of color with alkaline phosphatase-labeled anti-rabbit IgG and p-nitrophenyl phosphate as the substrate (Pierce, IL, USA). Specificity was calculated as follows: (experimental OD - background OD)/(total OD - background OD). Serum AF-AGE concentrations were read from the calibration curve obtained with the AF-AGEs-BSA standard and were expressed as AF-AGE units (U) per mL, with 1 U corresponding to 1.0 μg of AF-AGEs-BSA standard.

### Cell culture

HepG2 HCC cells were maintained in low glucose D-MEM supplemented with 10% FBS, 100 U/mL penicillin, and 100 µg/mL streptomycin at 37 °C in a humidified incubator under a 5% CO_2_ atmosphere. Cells were plated at a density of 1.5 × 10^4^ cells/cm^2^.

### Cell viability assay

The CellTiter-Glo luminescent cell viability assay was performed according to the manufacturer’s directions. In brief, cells were plated in triplicate into white opaque 96-well plates (Nunc, Roskilde, Denmark). After culture for 24 h, the cells were treated for 72 h with 1,5-AF or 1,5-AG reagent. (Cells were pretreated with aminoguanidine for 2 h before incubation with 1,5-AF reagent.) Then the cells were incubated for 10 min with CellTiter-Glo reagent, and measurement of luminescence was performed with a 96-well plate reader (GloMax-96 microplate luminometer; Promega). Background luminescence was measured in wells containing medium alone and was subtracted from values for the other wells. It was found that 1,5-AF, 1,5-AG, and aminoguanidine did not reduce luciferase activity, indicating no effect on cell viability assay.

### Slot blot (SB) analysis

Cells were washed by using PBS(−) without Ca^++^ or Mg^++^, and lysis was done in ice-cold buffer [2 M thiourea, 7 M urea, 4% CHAPS, 30 mM Tris, and EDTA-free protease inhibitor cocktail]. Then cell extracts were incubated on ice for 20 min and centrifuged at 10,000 × g at 4 °C for 15 min, after which the supernatant was collected as the cell extract. Protein concentrations were measured the Bradford method using BSA as the standard. For detection of AF-AGEs, equal amounts of cell extracts, the HRP-linked molecular marker, and AF-AGEs-BSA were all loaded onto polyvinylidene difluoride (PVDF) membranes (0.45 μm; Millipore, MA, USA) in the SB apparatus (Bio-Rad, CA, USA). Then each membrane was cut in half and blocked for 1 h at room temperature with 5% skim milk in PBS containing 0.05% Tween 20 (SM-PBS-T). After washing twice, membranes were incubated overnight at 4 °C with (1) the immunopurified anti-AF-AGE antibody (1:500) or (2) the neutralized immunopurified anti-AF-AGE antibody (a mixture of the immunopurified anti-AF-AGE antibody (1:500) and 40 µg/mL of AF-AGEs-BSA standard). Each membrane was then washed four times and incubated with the HRP-linked goat anti-rabbit IgG antibody (1:5,000) for 1 h at room temperature. After washing three times with PBS-T, membranes were moved into PBS. Immunoreactive proteins were detected with an ImmunoStar LD kit and band densities were measured using a Fusion FX fluorescence imager (M&S Instruments Inc., Osaka, Japan). The measured densities of the HRP-linked molecular marker bands were used to correct differences of density between membranes. The AF-AGEs content of each cell extract was calculated from the calibration curve created with the AF-AGEs-BSA standard.

### Statistical analysis

Stat Flex (ver. 6) software (Artech Co., Ltd., Osaka, Japan) was employed for all analyses. Results are expressed as the mean ± S.D. When multiple comparisons were performed, the significance of the differences of mean group values was assessed by a one-way analysis of variance followed by Tukey’s test. We used Student’s t-test when multiple comparison was not done. A probability <0.05 was considered to indicate significance.
